# Functional Characterization of Two Polymerizing Glycosyltransferases for the Addition of *N*-Acetyl-d-galactosamine to the Capsular Polysaccharide of *Campylobacter jejuni*

**DOI:** 10.1021/acs.biochem.4c00704

**Published:** 2025-01-24

**Authors:** Dao Feng Xiang, Tamari Narindoshvili, Frank M. Raushel

**Affiliations:** Department of Chemistry, Texas A&M University, College Station, Texas 77842, United States

## Abstract

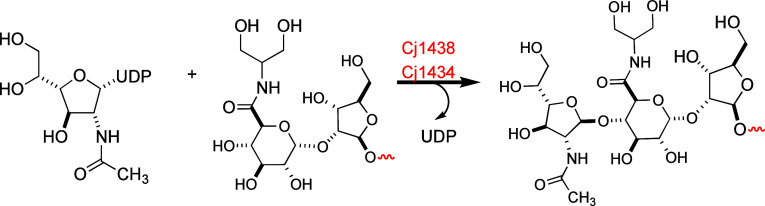

The exterior surface of the human pathogen *Campylobacter jejuni* is coated with a capsular polysaccharide (CPS) that consists of a repeating sequence of 2–5 different sugars that can be modified with various molecular decorations. In the HS:2 serotype from strain NCTC 11168, the repeating unit within the CPS is composed of d-ribose, *N*-acetyl-d-galactosamine, and a d-glucuronic acid that is further amidated with either serinol or ethanolamine. The d-glucuronic acid moiety is also decorated with d-glycero-l-gluco-heptose. Here, we show that two different GT2 glycosyltransferases catalyze the transfer of *N*-acetyl-d-galactosamine from UDP-NAc-d-galactosamine furanoside to the C4-hydroxyl group of the d-glucuronamide moiety at the growing end of the capsular polysaccharide chain. Catalytic activity was not observed with glycosides of d-glucuronic acid, and thus, the C6-carboxylate of the d-glucuronic acid moiety must be amidated prior to chain elongation. One of these enzymes comprises the N-terminal domain of Cj1438 (residues 1–325) and the other is from the N-terminal domain of Cj1434 (residues 1–327). These two glycosyltransferases are ∼87% identical in sequence, but it is not clear why there are two glycosyltransferases from the same gene cluster that apparently catalyze the same reaction. This discovery represents the second polymerizing glycosyltransferase that has been isolated and functionally characterized for the biosynthesis of the capsular polysaccharide in the HS:2 serotype of *C. jejuni*.

## Introduction

*Campylobacter jejuni* is an important human pathogen regarded as a major causative agent of bacterial gastroenteritis. The symptoms of *C. jejuni* infection typically include fever, nausea and abdominal cramping, and bloody diarrhea.^1^ Additionally, campylobacteriosis can result in myocarditis, irritable bowel syndrome, and Guillain-Barré Syndrome.^2−4^*C. jejuni* is transmitted to humans from animals, primarily chickens, where *C. jejuni* is part of the normal intestinal microbiota, but can also be transmitted from cattle, pigs, sheep, and domestic cats and dogs.^5,6^ Currently, there are no FDA-approved vaccines for the prevention of a *Campylobacter* infection. So far, the best candidates are vaccine conjugates, which mimic the surface exposed capsular polysaccharides (CPSs).^7^ The CPS protects bacteria from desiccation and from complement mediated phagocytosis.^8^ Additionally, the CPS of *C. jejuni* plays an important role in colonization and invasion of the host immune system.^9^

**Figure 1:**
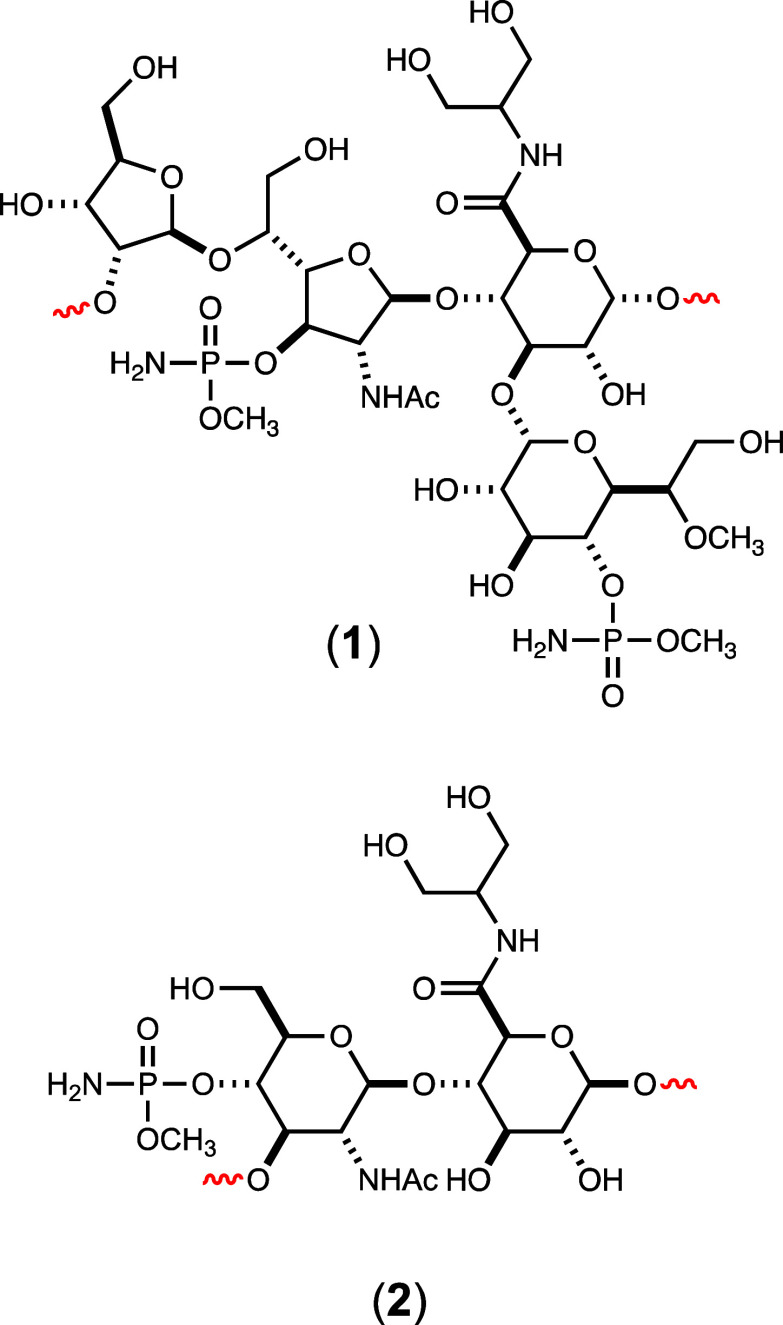
Structures of the repeating polysaccharides found in the CPS of the HS:2 serotype of *C. jejuni* strain NCTC 11168 (**1**) and the HS:19 serotype of *C. jejuni* strain NCTC 12517 (**2**).^10,13,14^ The glucuronic acid moiety in the HS:2 serotype can also be amidated with ethanolamine.^37^

The structure of the repeating CPS unit from *C. jejuni* NCTC 11168 (serotype HS:2) is illustrated in Figure 1 and is composed of d-ribose (d-Rib), *N*-acetyl-d-galactosamine (d-Gal*f*NAc), d-glucuronic acid (d-GlcA), and d-*glycero*-l-*gluco*-heptose.^10^ We and others have previously elucidated the biosynthetic pathways for some of the activated carbohydrates that are presumably used as sugar donors during the polymerization of this CPS.^10−39^ These include GDP-d-*glycero*-l-*gluco*-heptose, UDP-d-glucuronic acid (UDP-GlcA), and the furanose form of UDP-Gal*f*NAc.^18,21−24^ Previously, we identified and characterized the first enzyme (Cj1432) shown to catalyze the formation of the glycosidic bond between d-ribose and d-glucuronic acid using methyl-β-d-riboside as the acceptor substrate and UDP-GlcA as the sugar donor substrate.^40^ In this investigation, we have identified two additional enzymes (Cj1438 and Cj1434) that catalyze the formation of the glycosidic bond between *N*-acetyl-d-glucosamine and the d-glucuronic acid moiety during the polymerization of the CPS from the HS:2 serotype of *C. jejuni*.

## Materials and Methods

### Materials

Lysogeny broth (LB) and isopropyl-β-d-thiogalactopyranoside (IPTG) were purchased from Research Products International. HisTrap columns, HiTrap Q HP anion exchange columns, and Vivaspin 20 10 kDa MWCO spin filters were obtained from Cytiva. The 10K Nanosep spin filters were purchased from PALL Corp. (Port Washington, NY). All other materials and chemicals were purchased from Sigma-Aldrich, GE Healthcare, Bio-Sciences, or Carbosynth, unless otherwise stated.

### Synthesis of Potential Substrates for Cj1438_N_ and Cj1434_N_

**Figure 2:**
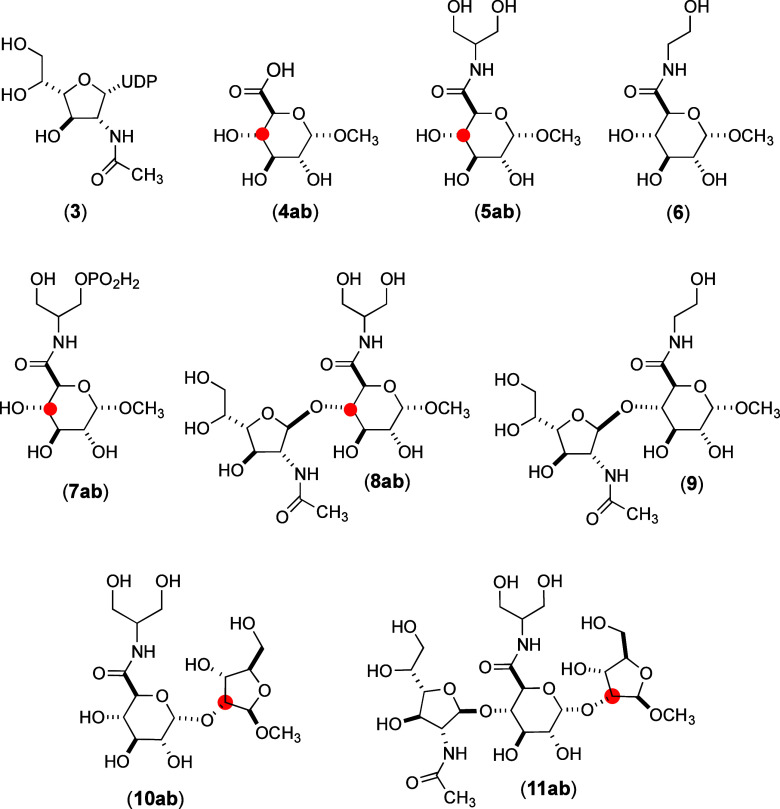
Structures of substrates and products produced for this investigation. The red dots in compounds **5**, **8**, **10**, and **11** represent a ^13^C-label at the indicated carbon. In these structures, the compounds denoted with an “**a**” are the unlabeled compounds, whereas the compounds denoted with a “**b**” are the ^13^C-labeled compounds.

Chemical syntheses of compounds **3**–**7** are described in the Supporting Information. The enzymatic syntheses of compounds **10a** and **10b** have been described previously.^40^ The structures of these compounds and other enzymatically formed products are listed in Figure 2.

### Cloning, Expression, and Purification of Cj1438_N_

The gene encoding the N-terminal domain (residues 1–325) of Cj1438 (UniProt id: Q0P8H6) from *C. jejuni* NCTC 11168 genomic DNA (ATCC 700819D-5) was purchased from Twist Biosciences in a pET28a expression vector with an N-terminal hexa-histidine tag. The purified protein is designated as Cj1438_N_ for this investigation. This construct was used to transform *Eschericha coli* BL21(DE3) competent cells, and single colonies were used to create starter cultures that contained kanamycin (50 μg/mL). The starter cultures were used to inoculate 1.0 L of LB. The cultures were allowed to grow at 37 °C until an OD_600_ of 0.6–0.8 was reached, and protein expression was induced by the addition of 1.0 mM IPTG. The cultures were grown at 20 °C for 18 h and then harvested by centrifugation (7000 rcf, 4 °C, 10 min). The resulting cell pellet was flash frozen in liquid nitrogen and stored at −80 °C. For purification of Cj1438_N_, 10 g of frozen cells was resuspended in 100 mL of 50 mM HEPES, 300 mM KCl, 10 mM imidazole, pH 8.0, containing 0.05 mg/mL of a protease inhibitor cocktail and 40 U/mL of DNase I. The resuspended cells were lysed by sonication (QSONICA Sonicator Ultrasonic Processor) in an ice bath. The lysate was clarified by centrifugation at 18,000 rcf at 4 °C for 30 min. The clarified supernatant fluid was passed through a 0.45 μm syringe filter (Whatman) and then loaded onto a 5 mL HisTrap column (GE Healthcare) attached to an NGC liquid chromatography system (BioRad) previously calibrated with binding buffer (50 mM HEPES, 300 mM KCl, and 10 mM imidazole, pH 8.0). The His-tagged protein was eluted with a 0–50% gradient of elution buffer (50 mM HEPES, 0.25 M KCl, and 0.50 M imidazole, pH 8.0). The fractions containing the desired protein, as identified by sodium dodecyl sulfate (SDS) gel electrophoresis, were combined, and the imidazole was removed from the protein solution by dialysis using a buffer containing 50 mM HEPES and 250 mM KCl, pH 8.0. The protein was concentrated to ∼10 mg/mL, aliquoted, frozen in liquid nitrogen, and stored at −80 °C. The concentration of the protein was determined spectrophotometrically using a computationally derived molar absorption extinction coefficient at 280 nm.^41^ The values of ε_280_ and molecular weight used for Cj1438_N_ were 43,130 M^–1^ cm^–1^ and 40,648 Da, respectively. About 47 mg of protein was obtained per liter of cell culture. The amino acid sequence of the purified protein is presented in Figure S1.

### Cloning, Expression, and Purification of Cj1434_N_

The gene encoding Cj1434 (UniProt ID: Q0P8I0) from *C. jejuni* NCTC 11168 was truncated (residues 1–327) and purchased from Twist Biosciences in a pET28a expression vector with an N-terminal hexa-histidine tag. The isolated protein is designated as Cj1434_N_ for this study. The plasmid containing the gene for Cj1434_N_ was transformed in *E. coli* BL21(DE3) competent cells. The conditions for expression and purification of Cj1434_N_ were the same as that for Cj1438_N_. The fractions eluted from the HisTrap column were pooled based on the SDS gel analysis results, concentrated to ∼3 mg/mL after the imidazole was removed by dialysis in 50 mM HEPES, 250 mM KCl, pH 8.0, frozen in liquid N_2_, and stored at −80 °C. The concentration of Cj1434_N_ was determined spectrophotometrically using a computationally derived molar absorption extinction coefficient at 280 nm.^41^ The values of ε_280_ and molecular weight used for Cj1434_N_ were 50,200 M^–1^ cm^–1^ and 41,035 Da, respectively. Approximately 22 mg of protein was isolated per liter of cell culture. The sequence of the purified protein is presented in Figure S1.

### Prediction of Three-Dimensional Structures of Cj1438_N_ and Cj1434_N_

The predicted three-dimensional structures of the full-length proteins, Cj1438 (AF-Q0P8H6–F1-v4) and Cj1434 (AF-Q0P8I0–F1-v4), were obtained from the AlphaFold database (https://alphfold.ebi.ac.uk).^42^ From these structural models, the predicted domain interfaces were used to help identify the truncation sites for the expression of soluble and presumably functionally active forms of the glycosyltransferases embedded within these multidomain proteins.

### Reactions Catalyzed by Cj1438_N_ and Cj1434_N_

The catalytic activities of Cj1438_N_ and Cj1434_N_ were initially investigated by adding the enzyme to 4.0 mM UDP-*N*-acetyl-d-glucosamine (**3**) with 3.0 mM potential acceptor substrates (compounds **4a**, **5a**, **6**, and **7a**). The reactions were conducted in 50 mM NH_4_HCO_3_, pH 8.0, 5.0 mM MgCl_2_, and either 20 μM Cj1438_N_ or Cj1434_N_. The reaction mixtures were incubated at 25 °C overnight (∼18 h). The protein was removed by using a 10K Nanosep spin filter (PALL) before product analysis by electrospray ionization (ESI)–mass spectrometry.

### Identification of Reaction Products

The products of the reaction catalyzed by Cj1438_N_ were investigated using ESI mass spectrometry in the positive ion mode. A 1.0 mL reaction mixture containing 20 μM Cj1438_N_, 4.0 mM substrate **3**, and 3.0 mM compound **5a** was incubated at 25 °C overnight. The reaction mixture was passed through a 10K Nanosep spin filter (PALL) to remove the protein, diluted to 15 mL, and then loaded onto a 5 mL HiTrap Q HP anion exchange column connected to an NGC Chromatography System (BioRad) and washed with water. The Cj1438_N_-catalyzed reaction product **8a** is neutral, so it was eluted in the wash step. The flow-through fractions were collected (5.0 mL each) and analyzed using mass spectrometry (Thermo Scientific Q Exactive Focus mass spectrometer). Fractions 2, 3, and 4 contained compound **8a**. The purest fractions (2 and 3) were pooled, lyophilized, dissolved in D_2_O, and analyzed using mass spectrometry and ^1^H NMR spectroscopy. Product **9** from the reaction of substrates **3** and **6** was also identified and purified using the same procedures as that for product **8a**.

### Identification of Additional Cj1438_N_-Catalyzed Products

The Cj1438_N_-catalyzed reaction products **11a** and **11b** were obtained from the incubation of substrate **3** with acceptor substrates **10a** and **10b**, respectively. The reaction conditions were the same as that for producing product **8a**, and the products were identified and purified using the same procedures as that for product **8a**.

### Reaction Rate Determinations for Cj1438_N_ and Cj1434_N_

The initial rates of the Cj1438_N_-catalyzed reactions were determined using a HiTrap Q HP anion exchange column to monitor the formation of UDP. Cj1438_N_ (40 μM) was incubated with substrate **3** (2.0 mM) and acceptor substrate **5a** (4.0 mM) in 50 mM NH_4_HCO_3_, pH 8.0, at 25 °C. An aliquot of the reaction mixture was heated at 100 °C for 60 s to quench the reaction at different time intervals. The precipitated protein was removed by centrifugation, and the supernatant fluid was passed through a 10K Nanosep spin filter (PALL), diluted in H_2_O, and then loaded to a 1.0 mL HiTrap Q HP anion exchange column. The column was connected to an NGC chromatography system (BioRad) (high-performance liquid chromatography) and washed with H_2_O. Substrate **3** and the product UDP were cleanly separated by ion exchange chromatography. The initial rate of the Cj1438_N_-catalyzed reaction was obtained based on the change in UDP concentration as a function of time. The catalytic reaction rate of Cj1438_N_ using substrate **3** as the donor substrate and acceptor substrates **6** and **10a** was also determined using the same procedures as described above for product **8a**. The reaction rates for the Cj1434_N_-catalyzed reactions were determined using the same procedures as those adopted for Cj1438_N_ except that the concentration of Cj1434_N_ used for substrate **10a** was 2.0 μM.

## Results and Discussion

### Search for Polymerizing Glycosyltransferases from *C. jejuni*

Within the gene cluster for the biosynthesis of the capsular polysaccharide in *C. jejuni*, serotype HS:2 (Figure S2), there are seven putative glycosyltransferases that could facilitate the biosynthesis of the CPS shown in Figure 1. Cj1431 was previously shown via genetic knockout experiments to be responsible for the transfer of d-*glycero*-l-*gluco*-heptose to the d-glucuronic acid moiety.^43^ We have shown previously that the N-terminal domain of Cj1432 is responsible for the transfer of d-GlcA from UDP-d-GlcA to the C2-hydroxyl group of d-ribose at the nonreducing end of the growing polysaccharide chain.^40^ We have also speculated that the C-terminal domain of Cj1432 is required for the transfer of d-ribose-5-P from phosphoribosyl pyrophosphate to the C5-hydroxyl group of Gal*f*NAc.^40,44^ However, the enzyme required for the transfer of d-Gal*f*NAc from UDP-Gal*f*NAc to the C4-hydroxyl group of the d-GlcA moiety of the growing polysaccharide is not known. Of the four remaining glycosyltransferases, the two enzymes most likely to catalyze this reaction are embedded in multidomain proteins Cj1438 and Cj1434.

### Deconstruction of Cj1438

**Figure 3:**
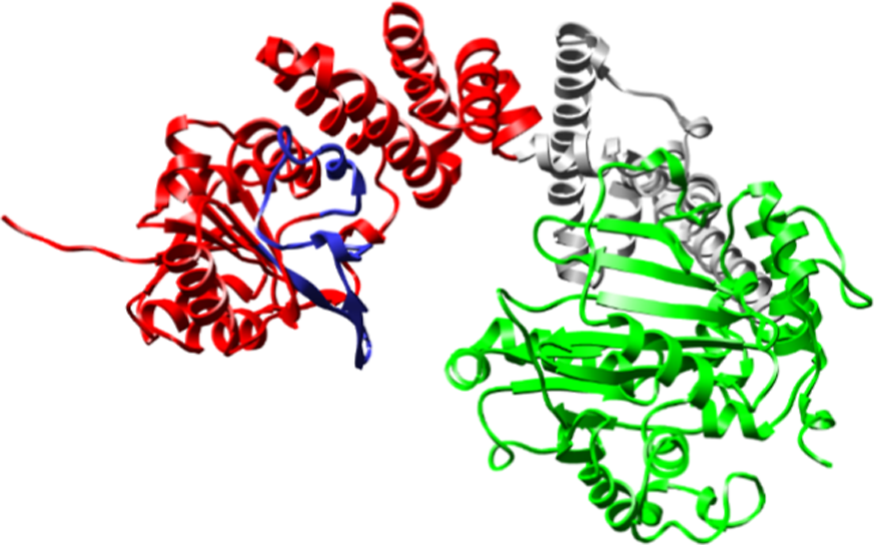
Predicted structure of Cj1438 using AlphaFold2.^42^ The red N-terminal domain is annotated to be a GT2 glycosyltransferase, and it extends from residues 1 to 325. The green C-terminal domain adopts a TupA-like ATP-grasp structural fold and extends from residues 453 through 776. These two domains are connected by a third domain colored gray (residues 326–452). The blue colored segment within the red colored domain represents the most diverged segment relative to that found within Cj1434_N_.

The three-dimensional structure of Cj1438 was predicted using AlphaFold2 and is presented in Figure 3.^42^ Cj1438 is composed of three individual domains. The N-terminal domain (colored red and now denoted as Cj1438_N_) comprises residues 1–325 and is functionally annotated as a GT2 glycosyltransferase by the CAZy database, and as a GT2 glycosyltransferase, it is expected to catalyze its reaction with inversion of configuration at C1 of the donor sugar.^45^ The C-terminal domain (colored green), which comprises residues 453–776, has a TupA-like ATP-grasp structural fold and has been shown previously by us to catalyze amide bond formation using the C6-carboxylate of the GlcA moiety and is denoted as Cj1438_C_.^38,39^ These two domains are connected to one another by a third domain (residues 326–452) of unknown function (colored gray in Figure 3). Based on these observations, attempts were made to express the N-terminal domain of Cj1438 via the utilization of a plasmid containing only the codons for the first 325 amino acids and an N-terminal polyhistidine purification tag. Cj1438_N_ was readily purified after heterologous expression in *E. coli.*

### Catalytic Properties of Cj1438_N_

**Figure 4:**
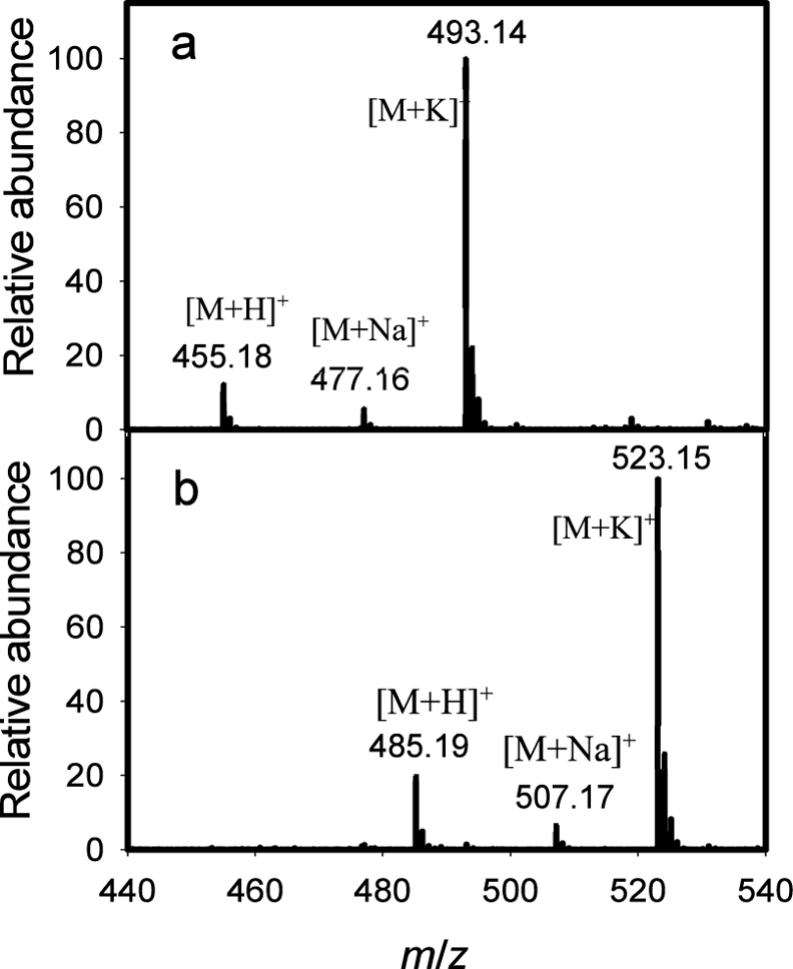
ESI mass spectra of products **8a** and **9** made from the catalytic activity of Cj1438_N_ with acceptor substrates **5a** and **6** using UDP-d-Gal*f*NAc (**3**) as the donor substrate. (a) ESI–MS of product **9**. (b) ESI–MS of product **8a**.

To test the proposal that the N-terminal domain of Cj1438 is responsible for the transfer of d-Gal*f*NAc to the growing polysaccharide chain, we incubated UDP-d-Gal*f*NAc (**3**) with a variety of possible acceptor substrates. These compounds included the α-methyl glycoside of d-glucuronic acid (**4a**) and three different amidated derivatives (**5a**, **6**, and **7a**). In this initial test of catalytic activity, only compounds **5a** and **6** were substrates for Cj1438_N_, forming products **8a** and **9**, respectively. The ESI mass spectra of the two isolated products are shown in Figure 4. The ESI-MS of product **9** (Figure 4a) exhibits *m*/*z* ratios of 455.16, 477.16, and 493.10 for the [M + H]^+^, [M + Na]^+^, and [M + K]^+^ ions, respectively, which match the calculated masses for these ions. For product **8a**, the ESI-MS (Figure 4b) exhibits *m*/*z* ratios of 485.19, 507.17, and 523.15 for the [M + H]^+^, [M + Na]^+^, and [M + K]^+^ ions, respectively. These results demonstrate that Cj1438_N_ catalyzes the transfer of d-Gal*f*NAc to the growing polysaccharide chain and that the d-GlcA moiety must be amidated prior to glycosyl transfer from UDP-d-Gal*f*NAc. The HSQC NMR spectrum of product **8a** is presented in Figure S3.

**Scheme 1:**
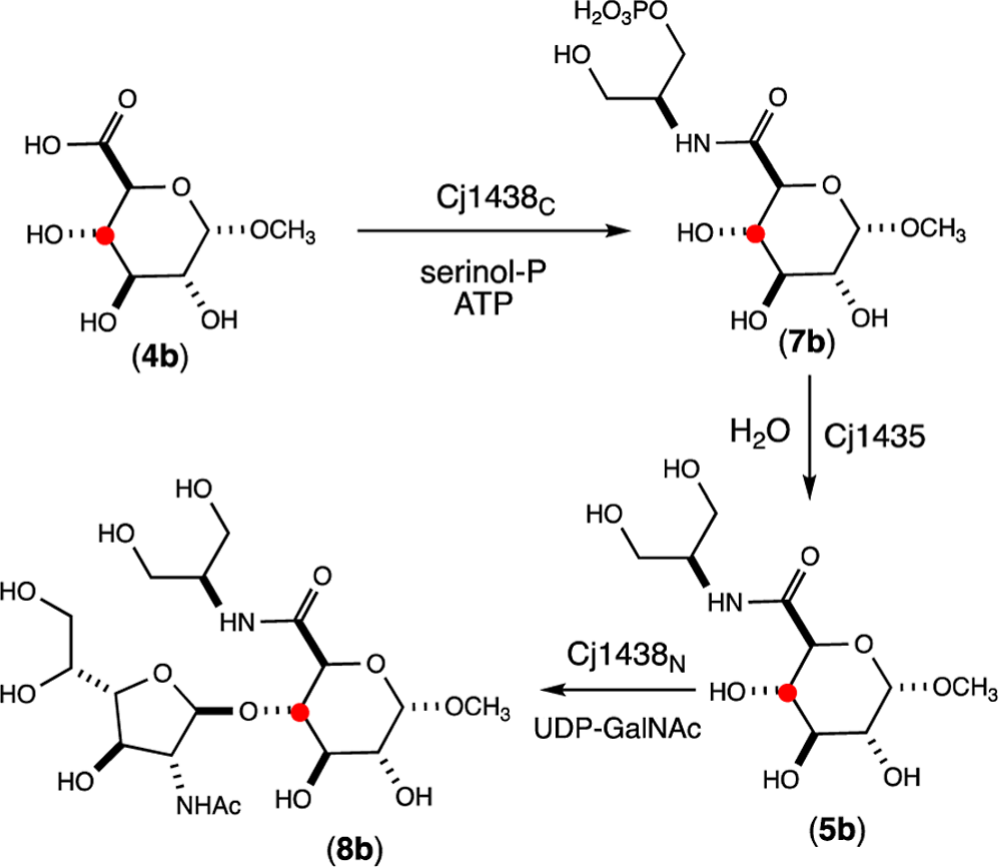
Enzymatic Preparation of ^13^C-Labeled Disaccharide **8b**

**Figure 5:**
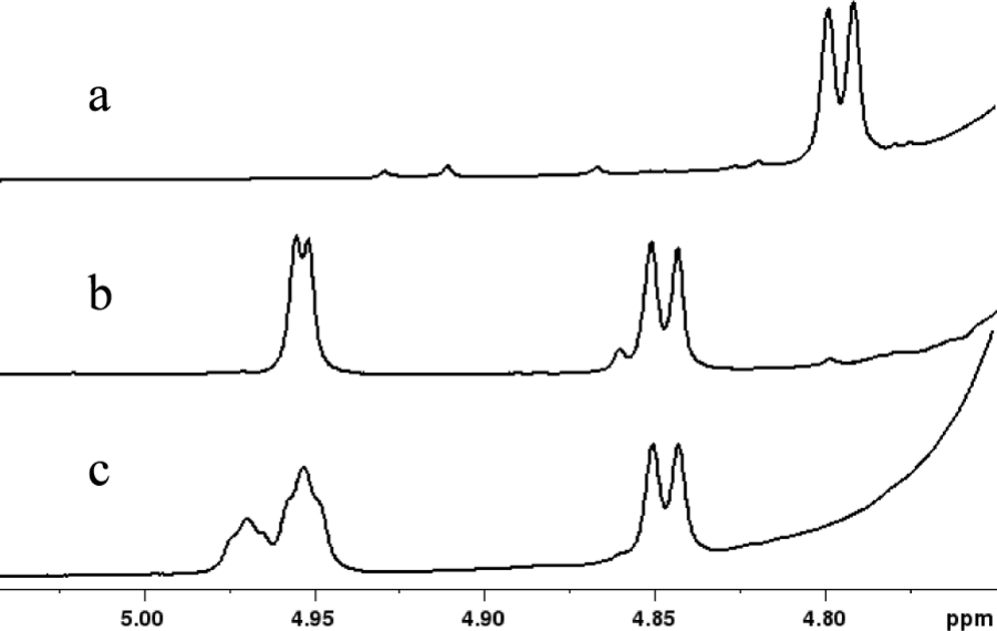
Portions of the NMR spectra for compounds **4b**, **8a**, and **8b** that highlight the resonances for the anomeric hydrogens. (a) Compound **4b**; (b) compound **8a**; (c) compound **8b**. Additional details are provided in the text.

To establish that the glycosidic bond between the d-glucuronamide and the d-Gal*f*NAc moieties catalyzed by Cj1438_N_ occurs with the hydroxyl group at C4 of the d-glucuronamide acceptor, the reaction was repeated with substrate **5b**, which contains a ^13^C-label at C4. Compound **5b** was enzymatically synthesized starting from compound **4b** as summarized in Scheme 1. Unfortunately, the methyl-d-glucuronic acid, **4b**, was isolated as a mixture of the α- and β-anomers in the ratio of 70:30, and this distribution of the two diastereomers is carried through in the enzymatically prepared **5b**. Compound **5b** was used with UDP-d-Gal*f*NAc in the presence of Cj1438_N_ to prepare ^13^C-labeled disaccharide **8b**. Portions of the ^1^H NMR spectra of the substrates and products showing the resonances for the anomeric hydrogens are presented in Figure 5. The anomeric proton for the α-anomer of the substrate **4b** resonates at 4.795 ppm, whereas the β-anomer (not shown) resonates at 4.350 ppm (Figure 5a). The unlabeled disaccharide **8a** exhibits resonances for the two anomeric hydrogens at 4.845 and 4.950 ppm. The doublet at 4.845 ppm originates from the d-glucuronamide moiety, while the doublet at 4.950 ppm is from the d-Gal*f*NAc moiety (Figure 5b). For the ^13^C-labeled product **8b**, the doublet for the anomeric hydrogen from the d-glucuronamide moiety is also observed at 4.845 ppm, while the resonance for the anomeric hydrogen from the GalNAc moiety is now an unresolved triplet at 4.950 ppm owing to the additional ^3^J_H–C_ coupling to the ^13^C-label at C4 of the glucuronamide moiety. The additional triplet at 4.970 ppm originates from the β-anomer. These results establish that the reaction catalyzed by Cj1438_N_ occurs with the C4 hydroxyl of the glucuronamide acceptor substrate.

**Scheme 2:**
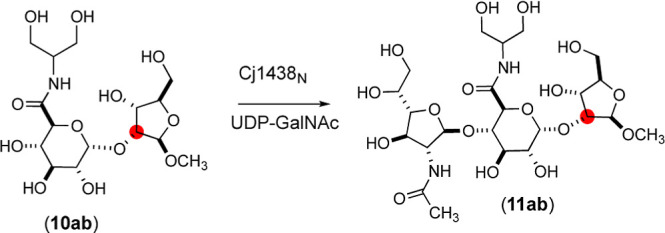
Reaction of Substrates **10a** and **10b** and UDP-GalNAc Catalyzed by Cj1438_N_

**Figure 6:**
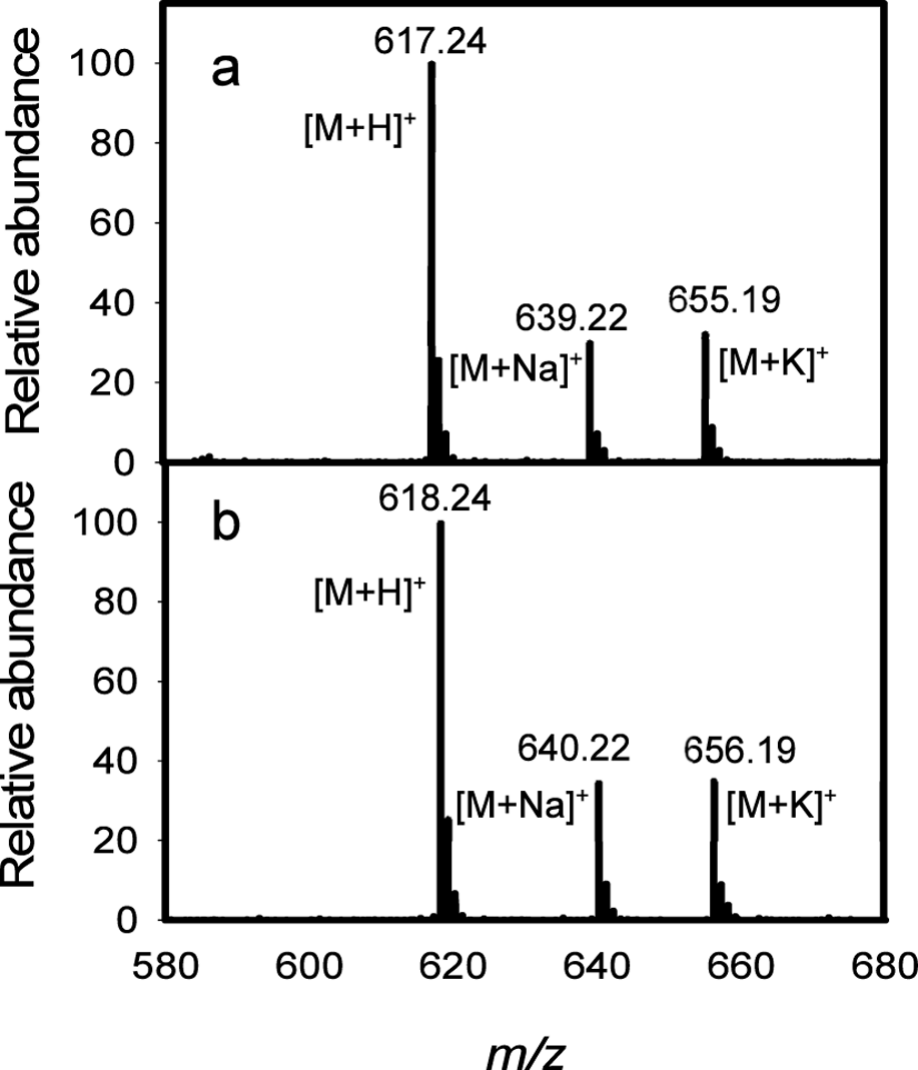
ESI–MS of products **11a** and **11b** made from the catalytic activity of Cj1438_N_ with acceptor substrates **10a** and **10b** using UDP-GalNAc (**3**) as the donor substrate. (a) ESI–MS of product **11a**. (b) ESI–MS of product **11b**.

To further elaborate the catalytic properties of Cj1438_N_, we tested this enzyme using d-ribose-d-glucuronamide disaccharides (**10a** and **10b**) as acceptor substrates to make trisaccharide products **11a** and **11b** (Scheme 2). Disaccharide **10b** contains a ^13^C-label at C2 within the d-ribose moiety of this substrate. The disaccharides **10a** and **10b** were excellent substrates for Cj1438_N_, and the ESI mass spectra of the isolated products are presented in Figure 6. Product **11a** exhibits *m*/*z* ratios of 617.24, 639.22, and 655.19, for the [M + H]^+^, [M + Na]^+^, and [M + K]^+^ complexes, respectively, which matched the calculated masses for these complexes. The ^13^C-labeled product **11b** exhibited *m*/*z* ratios of 618.24, 640.22, and 656.19 for the [M + H]^+^, [M + Na]^+^, and [M + K^+^]^+^ complexes that matched the expected values (Figure 6b).

### Deconstruction of Cj1434

**Figure 7:**
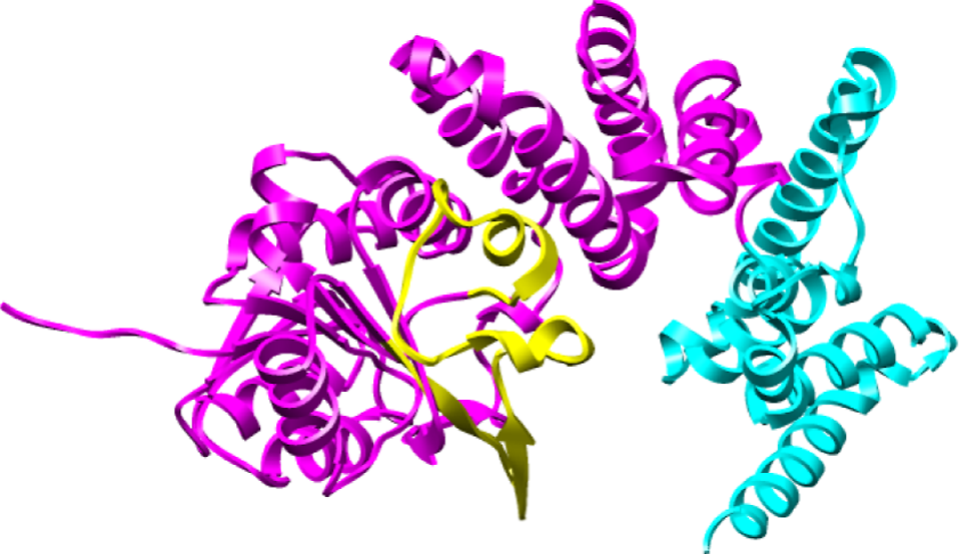
AlphaFold2-predicted structure of Cj1434. The N-terminal domain is highlighted in magenta and the C-terminal domain highlighted in cyan. The portion of the protein that differs most in sequence with the N-terminal domain of Cj1438 is highlighted in yellow.

**Figure 8:**
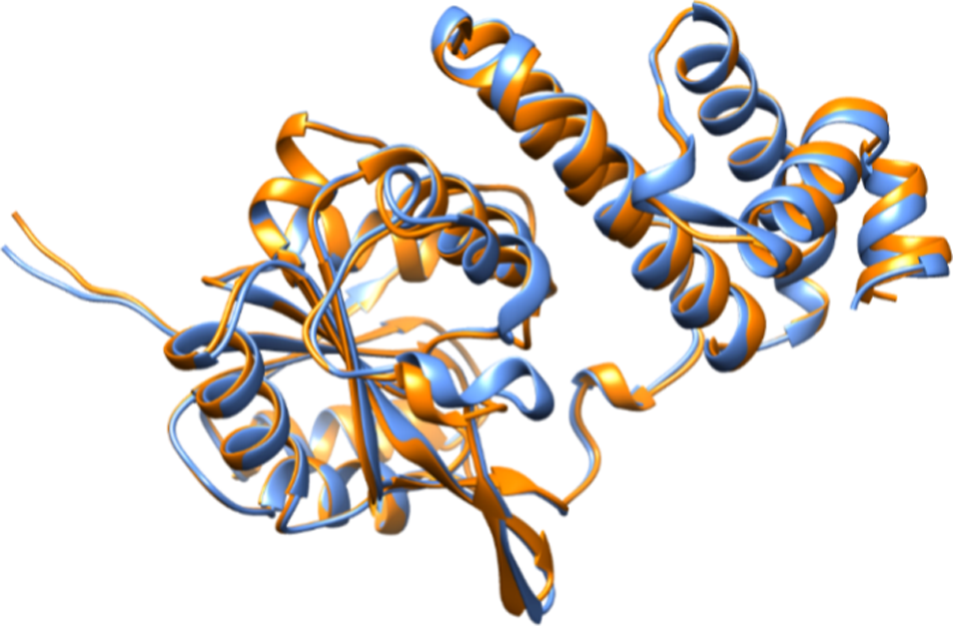
Structural alignment of the N-terminal domains of Cj1438 (orange) and Cj1434 (blue).

The GT2 glycosyltransferase Cj1434 is also a potential enzyme for the formation of a glycosidic bond between the d-GlcA and d-Gal*f*NAc moieties in the CPS of *C. jejuni*. The AlphaFold2-predicted structure of this enzyme is presented in Figure 7, where the first 327 residues are colored magenta and the C-terminal domain (residues 328–445) is shown in cyan. Curiously, the N-terminal domains of Cj1438 (residues 1–325) and Cj1434 (residues 1–327) are 87% identical in amino acid sequence (see Figure S4), suggesting that both enzymes will catalyze the same (or very similar) reactions. In Figure 8, a structural alignment of the N-terminal domains of Cj1434 and Cj1438 is presented, which illustrates the clear similarity in the two folded structures. We first attempted to express the gene for the entire sequence of Cj1434 but were unable to obtain soluble protein. Therefore, we obtained a plasmid that was limited to the expression for the first 327 amino acid residues. This protein was purified and subsequently denoted as Cj1434_N_.

The largest section of divergence in the amino acid sequences between Cj1434_N_ and Cj1438_N_ extends from residues 126 to 159, and these sections of the two proteins are highlighted in blue for Cj1438 in Figure 3 and in yellow for Cj1434 (Figure 7). Structural alignments of Cj1434_N_ and Cj1438_N_ with the N-terminal domain of the GT2 glycosyltransferase TarS from *Staphylococcus aureus* (PDB id: 5TZJ) bound with the donor substrate UDP-GlcNAc are presented in Figure S5. The predicted distances from C1 of the bound GlcNAc to the α-carbons of residues 126–159 in these two structural comparisons range from 14 to 29 Å. Therefore, this region of the two proteins is not involved **in** the binding of the sugar donor, UDP-Gal*f*NAc, but may contribute to the binding of an extended polysaccharide acceptor.

### Catalytic Properties of Cj1434N

The catalytic activities of Cj1434_N_ were found to be the same as that determined for Cj1438_N_. Using UDP-d-Gal*f*NAc (**3**) as the sugar donor, Cj1434_N_ was demonstrated to convert substrates **5a**, **6**, **10a**, and **10b** to products **8a**, **9**, **11a**, and **11b**, respectively. The ESI-MS results of the isolated products are presented in Figure S6.

### Reaction Rates for Catalysis by Cj1438_N_ and Cj1434_N_

The reaction rates for Cj1438_N_ were determined by using a HiTrap Q HP anion exchange column to measure the rate of formation of UDP as a function of time. Cj1438_N_ was incubated with donor substrate **3** (2.0 mM) and various acceptor substrates **5a**, **6**, and **10a** (4.0 mM). The reactions were quenched by denaturing the protein at different time intervals. The initial reaction rates using donor substrate **3** with acceptor substrates **5a**, **6**, and **10a** were determined to be 0.66 ± 0.05, 0.59 ± 0.02, and 1.14 ± 0.06 min^–1^, respectively. The reaction rates of Cj1434_N_ were determined using the same conditions and procedures as that for Cj1438_N_. The reaction rates of Cj1434_N_ using donor substrate **3** and acceptor substrates **5a**, **6**, and **10a** were determined to be 5.9 ± 0.3, 3.7 ± 0.2, and 204 ± 18 min^–1^, respectively. The time courses for product formation as a function of time are presented in Figure S7. Under these conditions, Cj1434_N_ is a better catalyst than Cj1438_N_.

**Figure 9:**
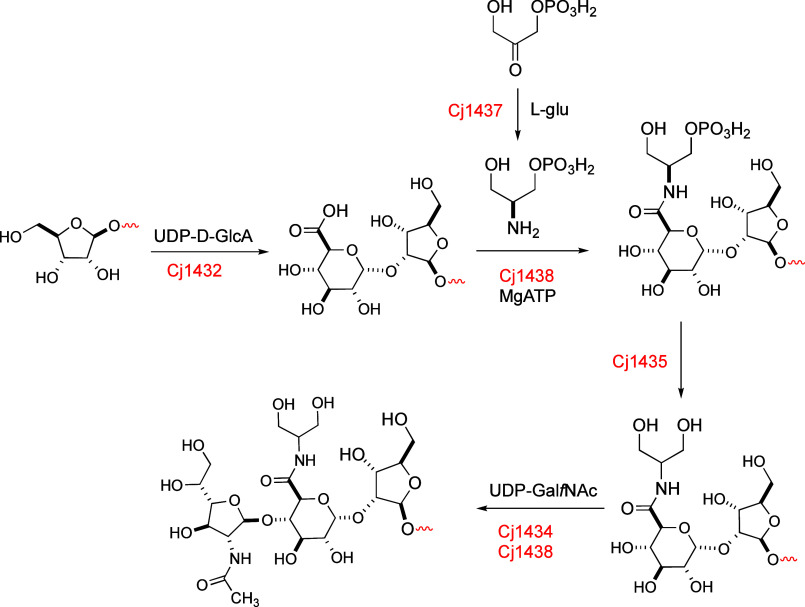
Summary of the reactions catalyzed by Cj1432, Cj1434, Cj1435, Cj1437, and Cj1438 during the biosynthesis of the capsular polysaccharide of the HS:2 serotype of *C. jejuni.*.

Previously, we demonstrated that the N-terminal domain of Cj1432 was able to catalyze the transfer of GlcA from UDP-GlcA to an acceptor d-ribofuranoside at C2 with retention of the configuration (40). In this report, we demonstrate that the N-terminal domains of either Cj1438 or Cj1434 can catalyze the transfer of Gal*f*NAc from UDP-Gal*f*NAc to a modified d-glucuronic acid acceptor substrate that must first be amidated with either serinol or ethanolamine. The amidation of the d-GlcA moiety has previously been shown to be catalyzed by the C-terminal domain of Cj1438 using ATP and either (*S*)-serinol-P or ethanolamine-P.^39^ The phosphoryl group is subsequently hydrolyzed by Cj1435.^38^ The collective reactions catalyzed by the two glycosyltransferases and the three auxiliary enzymes for the biosynthesis of the unique trisaccharide are summarized in Figure 9.

It is not at all clear why there are two enzymes that catalyze the transfer of Gal*f*NAc from UDP-d-Gal*f*NAc to the C4-hydroxyl group of the d-glucuronamide moiety of the growing carbohydrate chain. However, a similar situation is found in the gene cluster for the biosynthesis of the HS:19 serotype of *C. jejuni* (Figure S8). The CPS from the HS:19 serotype (compound **2**, Figure 1) is composed of a repeating disaccharide sequence of d-GlcNAc and the serinol amide of d-glucuronic acid.^33^ The C-terminal domain of HS19.11 (UniProt id: Q5M6M2) is homologous (61% identical) to the amidoligase domain from the C-terminal end of Cj1438 from HS:2 (Figure S9). The glycosyltransferase at the N-terminal domain of HS19.11 (now predicted to catalyze the transfer of d-GlcNAc from UDP-d-GlcNAc to the serinol amide of d-glucuronic acid) is 67% identical to the glycosyltransferase found in the N-terminal domain of HS19.08 (Figure S10).

We have now been able to characterize two different polymerizing glycosyltransferases (Cj1432_N_ and Cj1438_N_/Cj1434_N_) from the HS:2 serotype of *C. jejuni,* and this has enabled the chemoenzymatic synthesis of a trisaccharide composed of d-Gal*f*NAc-d-GlcA-d-Rib (product **11a**). The remaining polymerizing glycosyltransferase, needed for the attachment of d-ribose, will ultimately allow for the chemoenzymatic synthesis of the other two possible trisaccharides (d-Rib-d-Gal*f*NAc-d-GlcA and d-GlcA-d-Rib-d-Gal*f*NAc) in addition to the synthesis of much longer repeating polysaccharides. The characterization of the third polymerizing glycosyltransferase is in progress.

## Conclusions

The exterior surface of the human pathogen *C. jejuni* is coated with a capsular polysaccharide that helps to protect it from the host immune response. Different strains and serotypes of the bacterium synthesize unique and variable sequences of carbohydrates for the CPS. In the HS:2 serotype from *C. jejuni* NCTC 11168, the CPS is composed of a repeating sequence of d-ribose, *N*-acetyl-d-galactosamine, and d-glucuronic acid. The d-glucuronic acid moiety is further decorated by amidation with serinol and glycosylation with d-*glycero*-l-*gluco*-heptose. We identified two enzymes (Cj1438 and Cj1434) capable of catalyzing the transfer of *N*-acetyl-d-galactosamine from UDP-Gal*f*NAc to the C4-hydroxyl group of the d-glucuronamide moiety at the nonreducing end of the growing CPS. These results clearly demonstrate that after d-glucuronic acid has been added to the nonreducing end of the CPS, it must be first amidated with either serinol or ethanolamine by the catalytic activities of Cj1438 and Cj1435 prior to the addition of Gal*f*NAc. Cj1438 and Cj1434 are multidomain proteins, and the GT2 glycosyltransferase activities are located within the N-terminal half of these complex enzymes.

## Supporting Information Available

Amino acid sequences of the purified proteins, protein sequence comparisons, and NMR spectra of chemically synthesized substrates and enzyme-catalyzed reaction products (PDF)
