# Supplementary material for: Functional Characterization of Two Polymerizing Glycosyltransferases for the Addition of N-Acetyl-d-galactosamine to the Capsular Polysaccharide of Campylobacter jejuni
Source: Biochemistry. 2025 Jan 24;64(3):591–9. doi: 10.1021/acs.biochem.4c00704 (PMC11800379; doi:10.1021/acs.biochem.4c00704)
Supplement: Supplementary file 1 — bi4c00704_si_001.pdf [file bi4c00704_si_001.pdf]

SUPPLEMENTARY INFORMATION

Functional Characterization of Two Polymerizing Glycosyltransferases for the Addition of *N*-acetyl-D-Galactosamine to the Capsular Polysaccharide of *Campylobacter jejuni*

Dao Feng Xiang, Tamari Narindoshvili, and Frank M. Raushel*

Department of Chemistry, Texas A&M University,

College Station, TX 77842, US

*To whom correspondance may be addressed: raushel@tamu.edu

Synthesis of UDP-2-Acetamido-2-Deoxy-α-D-Galactofuranose (3). Synthesis of the ammonium salt of **3** was based on literature procedures.[1, 2] The synthetic precursor 2-acetamido-2-deoxy-α-D-galactofuranose-1-phosphate *bis*-triethylammonium salt **3a**, was synthesized as previously reported.[1] Compound **3a** was coupled with the morpholidate of UMP (**3b**) by modifying the conditions described in literature.[2] Compound **3a** (1.0 equiv.) was coupled with 3.0 equivalents of uridine 5'-phosphoromorpholidate dicyclohexylcarboxamidine (**3b**) using 3.5 equivalents of 4,5-dicyanoimidazole (DCI) as illustrate in **Scheme S1**.

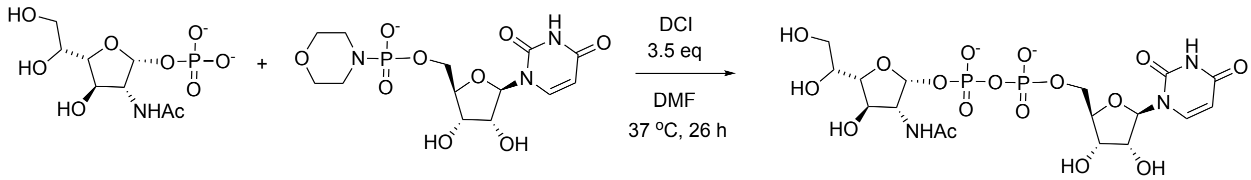

(**3a**) (**3b**) (**3**)

Scheme S1: Chemical synthesis of compound **3**.

To a solution of **3b** (59 mg, 0.086 mmol, 3.0 equiv) and **3a** (72%, 20 mg, 0.028 mmol, 1.0 equiv) in dry DMF (0.5 mL) was added 4,5-dicyanoimidazole (12.0 mg, 0.10 mmol, 3.5 equiv). The reaction was stirred at 37 °C for 26 h. The solution was then concentrated *in vacuo*. The residue was extracted with water (1 mL x 3), and the combined solution (1.5 mL) was frozen at -80 °C. Product **3** was purified using a 5-mL HiTrap™QHP anion exchange column with a linear gradient of 0% to 50 %, 500 mM NH_4OAc buffer (pH = 6.8). Fractions with the desired product were combined and lyophilized to obtain **3**.

^{1}H NMR (400 MHz, D_2O) δ 7.94 (d, J = 7.96 Hz, 1H), 5.99-5.94 (m, 2H), 5.64 (t, J = 4.8 Hz, 1H), 4.54-4.46 4 (m, 2H), 4.40–4.14 (m, 5H), 3.91 (dd, J_1 = 5.0 Hz, J_2 = 7.4 Hz, 1H), 3.81-3.74 (m, 1H), 3.71 (dd, J_1 = 4.2 Hz, J_2 = 11.8 Hz, 1H), 3.64 (dd, J_1 = 7.2 Hz, J_2 = 11.8 Hz, 1H), 2.07 (s, 3H) ppm.

^{31}P NMR (162 MHz, D_2O) δ −11.3 (d, J = 20 Hz, 1P), −12.9 (d, J = 20.0 Hz, 1P) ppm. (ESI-MS) *m/z* $[M - H]^-$ calc. for $C_{17}H_{26}N_3O_{17}P_2$: 606.0743; found: 606.0740.

Synthesis of Methyl-α-D-Glucuronic Acid (4).

Methyl-α-D-glucuronic acid (**4a**) was synthesized from methyl-α-D-glucopyranoside by catalytic oxidation (O_2/Pt/C) according to the procedure shown in **Scheme S2**.[3]

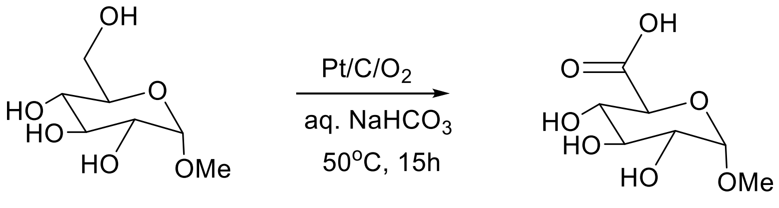

4ab

Scheme 2: Chemical synthesis of compounds **4a** and **4b**.

^1H NMR (400 MHz, D_2O) δ 4.81(d, J = 3.7 Hz, 1H), 3.89 (d, J = 10 Hz, 1H), 3.70 - 3.64 (m, 1H), 3.59 (dd, J_1 = 9.7 Hz, J_2 = 3.7 Hz, 1H), 3.52 - 3.46 (m, 1H), 3.42 (s, 3H).

(ESI-MS) *m/z* $[M - H]^-$ calc. for $C_7H_{11}O_7$: 207.0505; found: 207.0521.

Synthesis of α- and β-[$^{13}C_4$]-Methyl-D-Glucuronoside (7:3) (4b).

The mixture of α- and β- [4-^{13}C]-methyl-D-glucuronoside (7:3) was synthesized in two steps. D-[4-^{13}C] glucose was converted into a mixture of the α- and β-anomers (7:3) of [4-^{13}C]-methyl-D-glucoside in MeOH/AcCl (2.5 mL/0.1 mL) applying microwave (140 °C, 45 min). The obtained mixture was further converted by the above indicated oxidation procedure (*3*) to obtain α- and β- [$^{13}C_4$]-methyl-D-glucuroniside (7:3) (**4b**).

^1H NMR (400 MHz, D_2O) δ 4.81(d, J = 3.7 Hz, 0.7H), 4.37(d, J = 8.0 Hz, 0.3H), 3.92-3.86 (m, 0.6H), 3.74 - 3.64 (m, 1.4H), 3.60 (dd, J_1 = 9.7 Hz, J_2 = 3.4 Hz, 0.7H), 3.57(s, 0.9H). 3.52 - 3.46 (m, 0.45H), 3.42 (s, 2.1H), 3.33-3.26(m, 0.85H).

Synthesis of Compound 5. Compound **5** was synthesized from commercially available 2,3,4-tribenzyl protected methyl-α-D-glucopyranoside (**Scheme S3**) according to previously described procedures.[4-7]

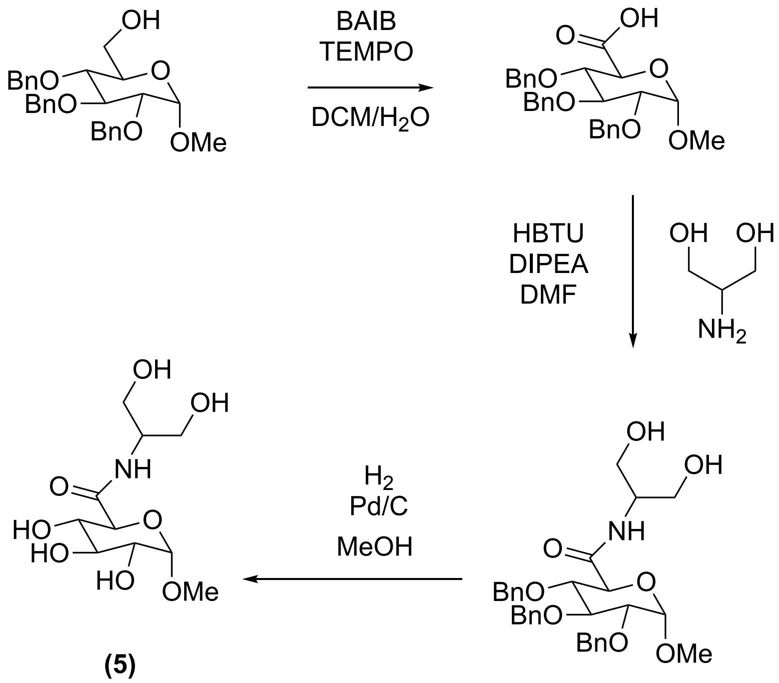

(5)

Scheme S3: Chemical synthesis of compound **5**.

Compound **5:** ^{1}H NMR (400 MHz, D_2O) δ 4.87 (d, J = 3.7 Hz, 1H), 4.10-4.01 (m, 2H), 3.74 - 3.60 (m, 6H), 3.57 (t, J = 9.4 Hz, 1H), 3.43 (s, 3H).

^{13}C NMR (125 MHz, D_2O) δ 171.4, 99.8, 72.7, 71.6, 71.4, 70.9, 60.5, 55.6, 52.9 49.0, 38.1. HRMS (ESI$^+$) *m/z* [M + H]$^+$ calc. for $C_{10}H_{20}NO_8$: 282.1183; found: 282.1181.

Synthesis of Compound 6. Compound **6** was synthesized from commercially available 2,3,4-tribenzyl protected methyl-α-D-glucopyranoside (**Scheme S4**) according to described procedures.[4-7]

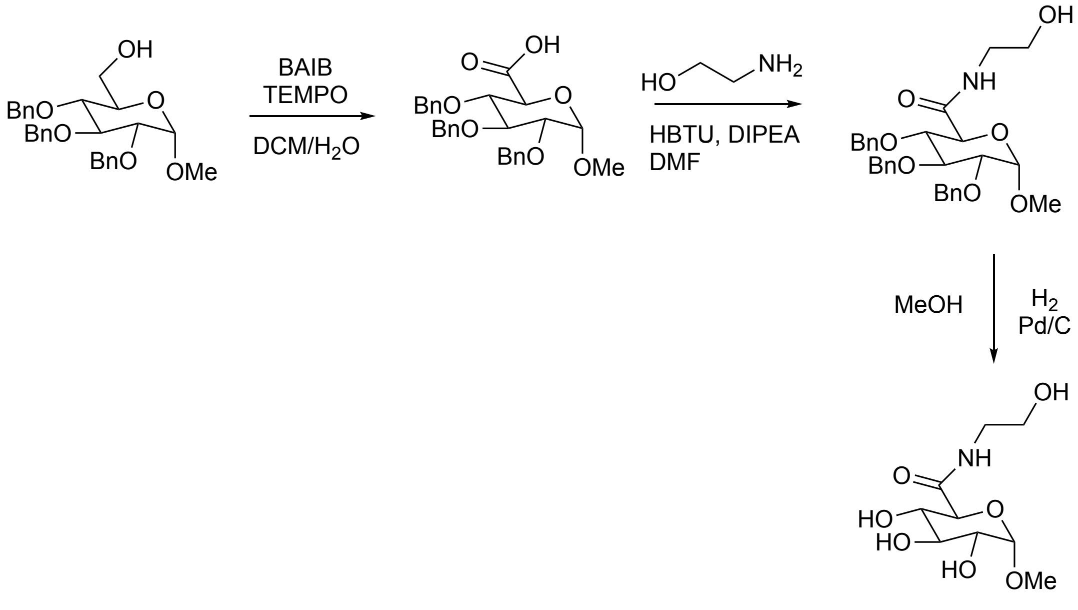

(6)

Scheme S4: Chemical synthesis of compound **6**.

Compound 6: ^{1}H NMR (400 MHz, D_2O) δ 4.86(d, J = 3.7 Hz, 1H), 4.04 (d, J = 10.0 Hz, 1H), 3.70 - 3.65 (m, 3H), 3.61 (dd, J_1 = 10.0 Hz, J_2 = 3.7 Hz, 1H), 3.52 - 3.52 (m, 1H), 3.42 (s, 3H), 3.42-3.36 (m, 2H).

^{13}C NMR (125 MHz, D_2O) δ 171.3, 99.8, 72.7, 71.6, 71.4, 70.9, 59.9, 55.5, 48.9, 41.4. HRMS (ESI$^+$) m/z $[M + H]^+$ calc. for $C_9H_{18}NO_7$: 252.1078; found: 252.1075

Synthesis of Compound 7. Compound **7** was synthesized from commercially available 2,3,4-tribenzyl protected methyl-α-D-glucopyranoside (**Scheme S5**) according to previously described procedures.[4-7].

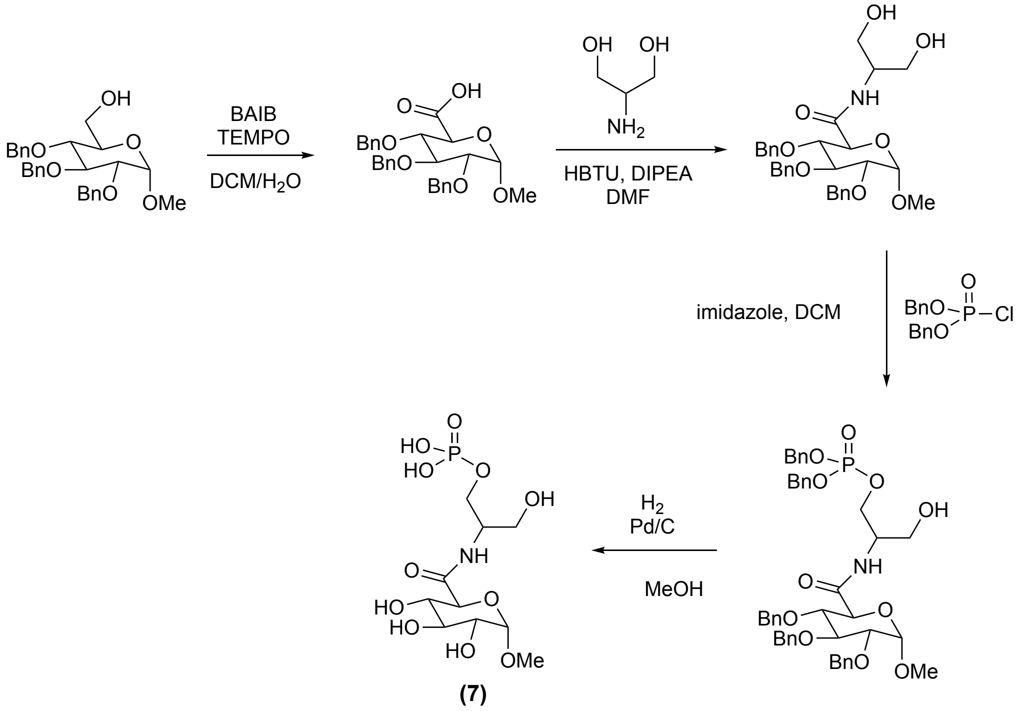

Scheme S5. Chemical synthesis of compound **7**.

Compound 7: ^{1}H NMR (400 MHz, D_2O) δ 4.78 (d, J = 3.5 Hz, 1H), 4.22-4.13 (m, 1H), 4.12-4.06 (m, 2H), 4.00 (d, J = 9.8 Hz, 1H) 3.73 - 3.64 (m, 3H), 3.53-3.46 (m, 2H), 3.43 (s, 3H).

^{13}C NMR (125 MHz, D_2O) δ 171.4, 99.8, 72.7, 71.6, 71.4, 70.9, 60.5, 55.6, 52.9 49.0, 38.1. HRMS (ESI$^-$) m/z [M - H]$^-$ calc. for $C_{10}H_{19}NO_{11}P$: 360.0690, found: 360.0704.

Amino acid sequence of full length Cj1438 (UniProt id: Q0P8H6):

MMNYNTPKVSIVVPSLNSISYIRECIDSILNQTLKDIEILCIDANSTDGTLEVLKNYEKKDKRLRVIISDKKSYGYQMNLGIKEA
KGEYLGIVESDDYIKTNMYERLYEIAKKNDCEVVKGDFYILESNKGKYSKITPIDFLYNQIISFKTHPNIFNFQSINPIGIYRLD
LLRTNQIKLNETPGASYQDNGLWFQIFALAKSIYFINEAFYMLRRDNPNSSVKSKEKVYCACEEYDFIRDFLKKHPDLEKTLAPI
CALHRFGNYMFTLERIDERYKLDFLKRFSQDFRKILKDKELDENLFGDGDMKIIYSIVENPENYYFLYMGYCNDMFGKLYFGASE
RIKWQLSYRIGKLLIDLKNPVQILKFPFKLFLEIKQFKFEQKIYKTTIKFYPNLQLPPLEEYSDYEQALKTKKHLSYILGKSFIN
NPILFIFKIKKIYKQYKKDISSSKKNIKELSDYDFLLNRHKQIFDYTPDFKCPVTFNEKLIYRILYDRSCIYSFLADKIKMRFYV
ASALSDNHEYSWDKIDILNEKSILFNNIDDLQDKIFETNKCKYLPKIYGIYKNIYDINFNELPNSFVLKTNHDCGGYVIVENKQE
FLRDTVVFSNAMKKLKKHLEWNYYSVFREWHYKDIEPRVFAEELLLGENKKPADTYKFHIFDKENLSNNFIQVTTDRFDNYQRAM
FDLSWNLAPFNFMYDNKNVTMIPKKPNLLDSMINISLILAKPFDYVRVDLYQFDKKIYIGELTFTHGAAGEKVIPKEWDKKLGDL
WRLKRLDNASK

Amino acid sequence of purified truncated Cj1438$_N$ (Cj1438$_{1\text{-}325}$).

MGSSHHHHHHSSGLVPRGSHMMNYNTPKVSIVVPSLNSISYIRECIDSILNQTLKDIEILCIDANSTDGTLEVLKNYEKKDKRLR
VIISDKKSYGYQMNLGIKEAKGEYLGIVESDDYIKTNMYERLYEIAKKNDCEVVKGDFYILESNKGKYSKITPIDFLYNQIISFK
THPNIFNFQSINPIGIYRLDLLRTNQIKLNETPGASYQDNGLWFQIFALAKSIYFINEAFYMLRRDNPNSSVKSKEKVYCACEEY
DFIRDFLKKHPDLEKTLAPICALHRFGNYMFTLERIDERYKLDFLKRFSQDFRKILKDKELDENLFGDGDMKIIYSIVENPENYY
FLYMG

Amino acid sequence of full length Cj1434 (UniProt id: Q0P8I0):

MMNYNTPKVSIVVPSLNSISYIRECIDSILNQTLKDIEILCIDANSTDGTLEVLKNYEKKDKRLRVIISDKKSYGYQMNLGIKEA
KGEYLGIVESDDYIKTNMYERLYEIAKKNDCEVVKGDFYIFAYGKTEYVNVLRNSCEDIYNYKVNWNKDIRIFLGSDGINPIGIY
RLDLLRTNQIKLNETPGASYQDNGLWFQIFALAKSIYFINEAFYMLRRDNPNSSVKSKEKVYCACEEYDFIRDFLKKHPDLEKTL
APICALHRFGNYMFTLERIDERYKLDFLKRFSQDFRKILKDKELDENLFGNINMQRINKIIENPVIYYYFSRGARARLQNQLVYR
LGKVVVEAKSFNKIIKLPFLMLKICLEHNFEHKVYRSIVQFRPDLKLLPLECYLDYHEALVIKEHLSYKFGKLILLSFKGWYKGK
IFILPFMLKKRYKEYKNKMI

Amino acid sequence of purified truncated Cj1434$_N$ (Cj1434$_{1\text{-}327}$)

MGSSHHHHHHSSGLVPRGSHMMNYNTPKVSIVVPSLNSISYIRECIDSILNQTLKDIEILCIDANSTDGTLEVLKNYEKKDKRLR
VIISDKKSYGYQMNLGIKEAKGEYLGIVESDDYIKTNMYERLYEIAKKNDCEVVKGDFYIFAYGKTEYVNVLRNSCEDIYNYKVN
WNKDIRIFLGSDGINPIGIYRLDLLRTNQIKLNETPGASYQDNGLWFQIFALAKSIYFINEAFYMLRRDNPNSSVKSKEKVYCAC
EEYDFIRDFLKKHPDLEKTLAPICALHRFGNYMFTLERIDERYKLDFLKRFSQDFRKILKDKELDENLFGNINMQRINKIIENPV
IYYYFSR

Figure S1. Amino acids sequences of the two purified proteins used for this investigation (Cj1438$_N$ and Cj1434$_N$). Those amino acids colored in red font are for the poly-histidine purification tag.

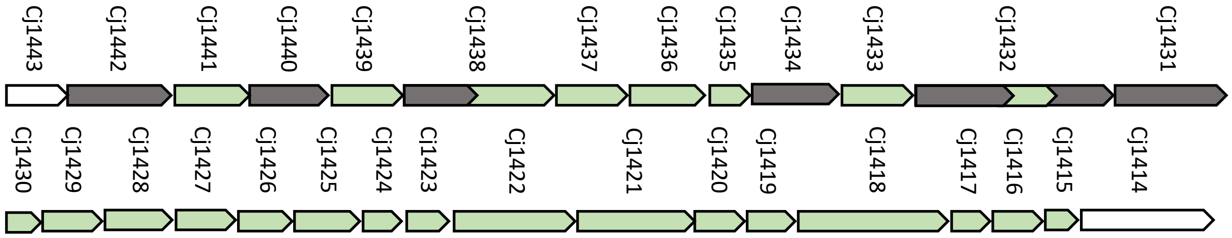

Figure S2: Gene cluster for the biosynthesis of the capsular polysaccharide from *C. jejuni* NCTC 11168 (serotype HS:2). The genes colored gray are for the 7 putative glycosyltransferases required for the biosynthesis of the capsular polysaccharide.

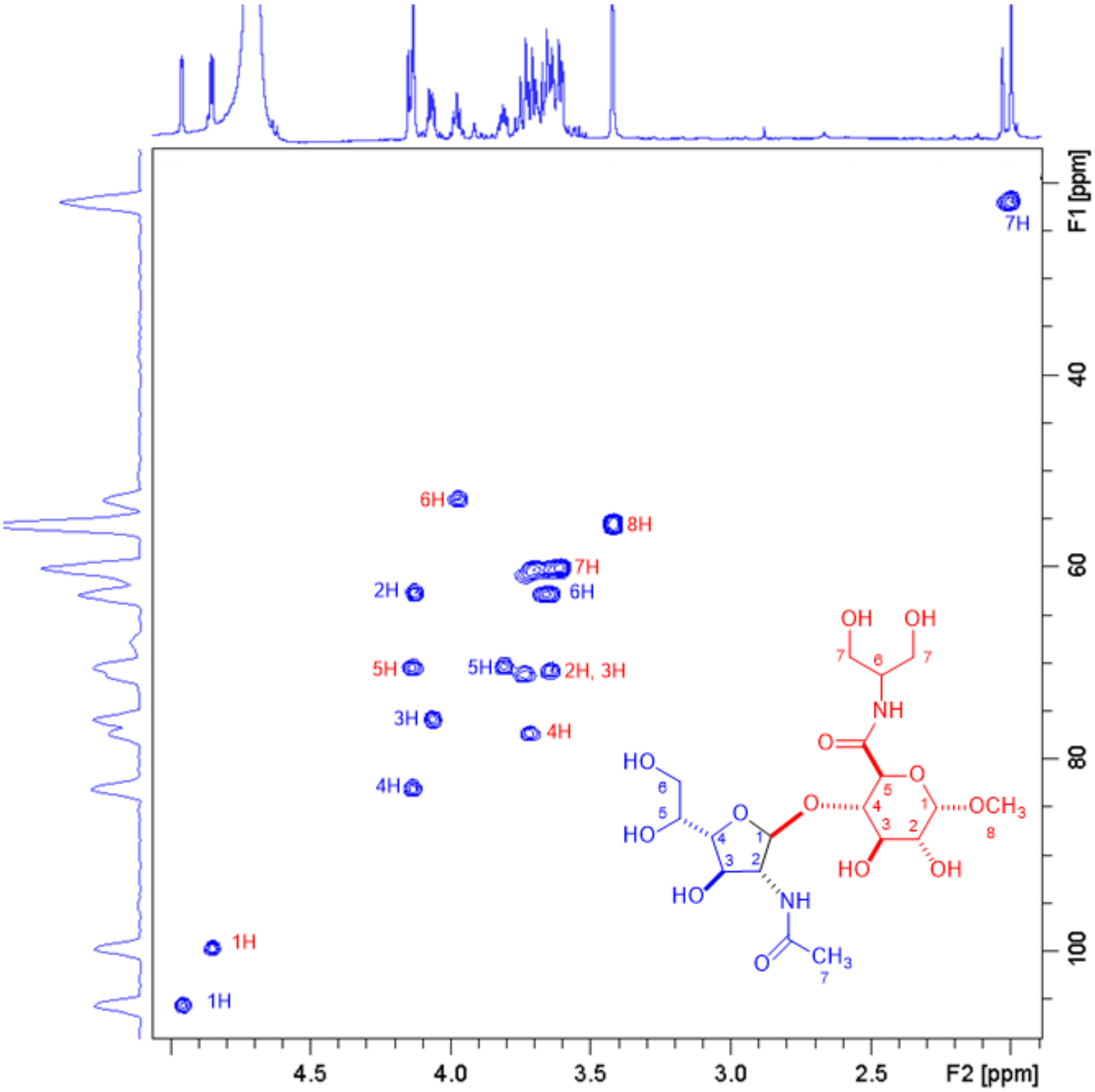

Figure S3: HSQC NMR spectrum of product **8a**.

```
Cj1438     1 MMNYNTPKVSIVVPSLNSISYIRECIDSILNQTLKDIEILCIDANSTDGTLEVLKNYEKK  60
             MMNYNTPKVSIVVPSLNSISYIRECIDSILNQTLKDIEILCIDANSTDGTLEVLKNYEKK
Cj1434     1 MMNYNTPKVSIVVPSLNSISYIRECIDSILNQTLKDIEILCIDANSTDGTLEVLKNYEKK  60

Cj1438    61 DKRLRVIISDKKSYGYQMNLGIKEAKGEYLGIVESDDYIKTNMYERLYEIAKKNDCEVVK  120
             DKRLRVIISDKKSYGYQMNLGIKEAKGEYLGIVESDDYIKTNMYERLYEIAKKNDCEVVK
Cj1434    61 DKRLRVIISDKKSYGYQMNLGIKEAKGEYLGIVESDDYIKTNMYERLYEIAKKNDCEVVK  120

Cj1438   121 GDFYILESNKGKYSKI--TPIDFLYNQIISFKTHPNIF-NFQSINPIGIYRLDLLRTNQI  177
             GDFYI    K +Y  +     + +YN  +++     IF     INPIGIYRLDLLRTNQI
Cj1434   121 GDFYIFAYGKTEYVNVLRNSCEDIYNYKVNWNKDIRIFLGSDGINPIGIYRLDLLRTNQI  180

Cj1438   178 KLNETPGASYQDNGLWFQIFALAKSIYFINEAFYMLRRDNPNSSVKSKEKVYCACEEYDF  237
             KLNETPGASYQDNGLWFQIFALAKSIYFINEAFYMLRRDNPNSSVKSKEKVYCACEEYDF
Cj1434   181 KLNETPGASYQDNGLWFQIFALAKSIYFINEAFYMLRRDNPNSSVKSKEKVYCACEEYDF  240

Cj1438   238 IRDFLKKHPDLEKTLAPICALHRFGNYMFTLERIDERYKLDFLKRFSQDFRKILKDKELD  297
             IRDFLKKHPDLEKTLAPICALHRFGNYMFTLERIDERYKLDFLKRFSQDFRKILKDKELD
Cj1434   241 IRDFLKKHPDLEKTLAPICALHRFGNYMFTLERIDERYKLDFLKRFSQDFRKILKDKELD  300

Cj1438   298 ENLFGDGDMKIIYSIVENPENYYFLMG  324
             ENLFG+ +M+ I  I+ENP  YY+
Cj1434   301 ENLFGNINMQRINKIIENPVIYYYFSR  327
```

Figure S4: Sequence comparison between Cj1438$_N$ (UniProt id: Q0P8H6) and Cj1434$_N$ (UniProt id: Q0P8I0). The overall sequence identity is 87%.

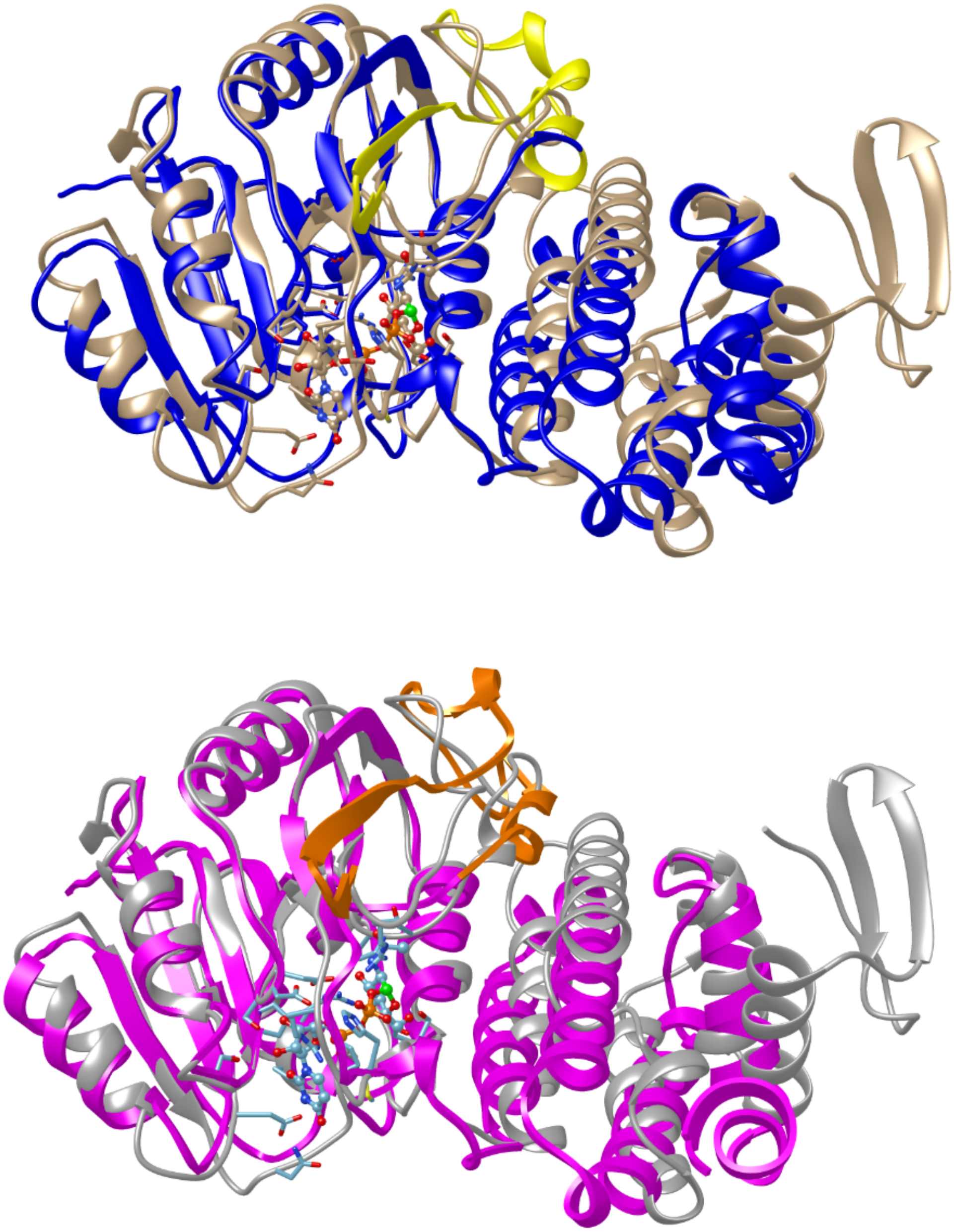

Figure S5. Superimposition of the GT2 glycosyltransferase TarS from *Staphylococcus aureus* (PDB id: 5TZJ) bound with the donor substrate UDP-GlcNAc and the AlphaFold calculated structures of $Cj1434_N$ (blue, Panel A) and that of $Cj1438_N$ (purple, Panel B). The loop containing residues 126-159 of $Cj1434_N$ is highlighted in yellow and the loop containing residues 126-159 from $Cj1438_N$ is highlighted in orange. The UDP-GlcNAc bound in the active site of TarS is shown in a ball and stick format.

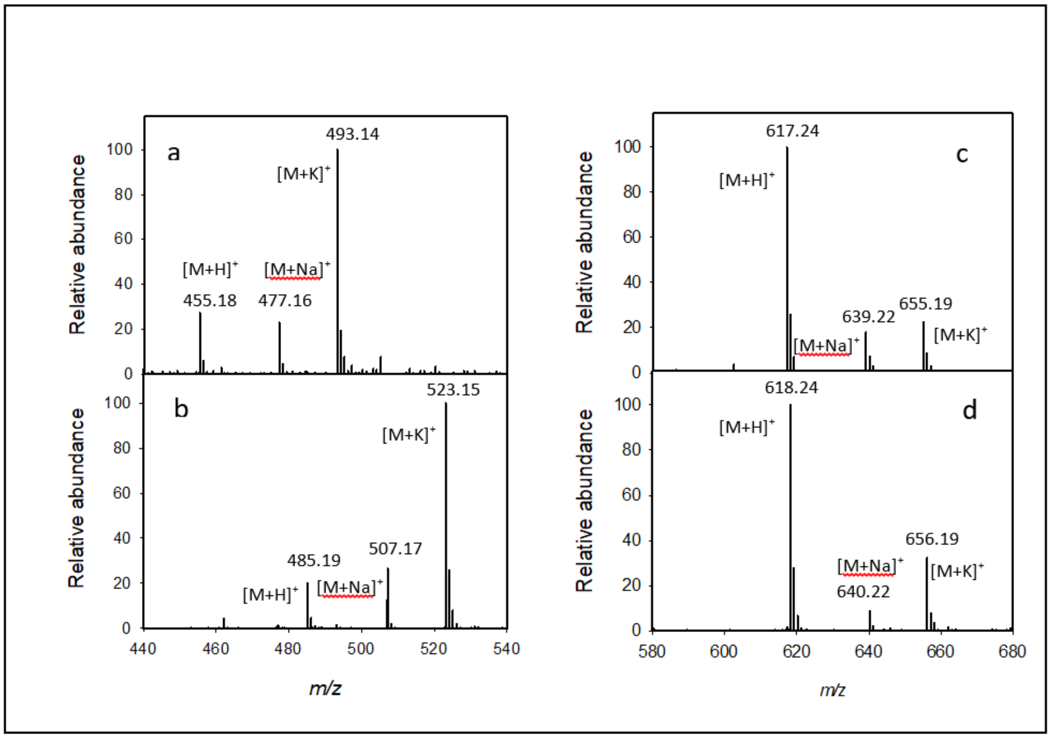

Figure S6: ESI-MS of product 8a (panel a); product **9** (panel b); product **11a** (panel c); and product **11b** (panel d) formed by the catalytic activity using Cj1434 as the glycosyltransferase.

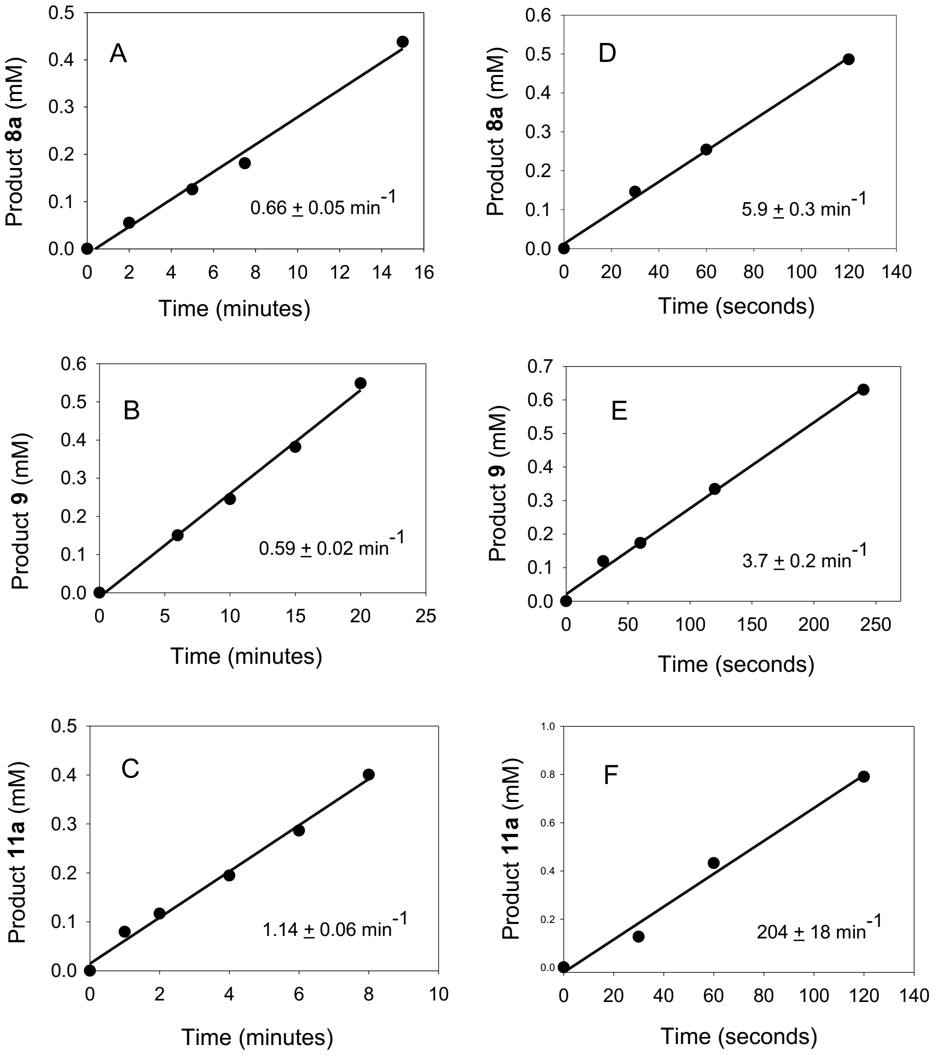

Figure S7. Time course for determining the initial reaction rate for $Cj1438_N$ and $Cj1434_N$. Reactions of 2.0 mM donor substrate **3** and 4.0 mM acceptor substrates **5a**, **6**, and **10a** with 40 μM $Cj1438_N$ are shown in panels **A**, **B**, and **C**, respectively. Reactions of 2.0 mM donor substrate **3,** and 4.0 mM acceptor substrates **5a** and **6** with 40 μM $Cj1434_N$ are shown in panels **D** and **E**, respectively. Reaction of 2.0 mM donor substrate **3,** and 4.0 mM acceptor substrate **10a** with 2.0 μM Cj1434N is shown in Panel F.

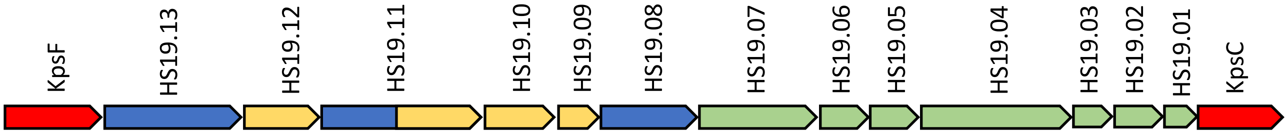

Figure S8: Gene cluster for the biosynthesis of the capsular polysaccharide in the HS:19 serotype of *C. jejuni*. The genes depicted in green are those for the biosynthesis and phosphoryl transfer for the phosphoramidate modification to the CPS. Those depicted in blue are likely glycosyltransferases. Those depicted in tan are those required for the biosynthesis of the serinol amide with glucuronic acid.

```
Cj1438   460 FLLNRHKQIFDYTPDFKCPVTFNEKLIYRILYDRSCIYSFLADKIKMRFYVASALSDNHE 519
             F   RHK IF+Y PDFK P TFNEKL++R+LYDRS +Y+FLADK+KMR ++   LS
HS19.11  523 FFKERHKAIFNYIPDFKHPQTFNEKLVFRMLYDRSPLYTFLADKLKMRIFIQQILS---- 578

Cj1438   520 YSWDKIDILNEKSILFNNIDDLQDKIFETNKCKYLPKIYGIYKNIYDINFNELPNSFVLK 579
               +D+ +I +  S+LF +ID +QDKI  TN C+YLPK+Y IY +IYDI+F+ LP SFVLK
HS19.11  579 -QFDESNIFDNNSVLFQDIDKIQDKILNTNICEYLPKLYAIYDDIYDIDFDILPESFVLK 637

Cj1438   580 TNHDCGGYVIVENKQEFLRDTVVFSNAMKKLKKHLEWNYYSVFREWHYKDIEPRVFAEEL 639
             TNHDCGGYVIVE+K +FLRD  +FS++M+KL  HL  NYY + REWHYKDI+P++FAEEL
HS19.11  638 TNHDCGGYVIVEDKIKFLRDIDLFSSSMQKLHNHLHSNYYYLSREWHYKDIKPKIFAEEL 697

Cj1438   640 LLGENKKPADTYKFHIFDKENLSNNFIQVTTDRFDNYQRAMFDLSWNLAPFNFMYDNKNV 699
             L+ +N K ADTYKFHIFD +NL+NN+IQVTTDRF+NYQR + D +WN+APFNF Y+  +
HS19.11  698 LIDKNGKLADTYKFHIFDHKNLNNNYIQVTTDRFNNYQRFIMDSNWNIAPFNFTYE-VSK 756

Cj1438   670 TMIPKKPNLLDSMINISLILAKPFDYVRVDLYQFDKKIYIGELTFTHGAAGEKVIPKEWD 729
               +P +P+  + M  ISL L+K FDYVRVDLY  D +IYIGELTFTHGAAGEK+ P  WD
HS19.11  757 DKLPNRPSEFEKMFEISLKLSKMFDYVRVDLYCIDNRIYIGELTFTHGAAGEKLNPNCWD 816

Cj1438   730 KKLGDLWRLKRLDNASK  746
             KKLG LW +++L + +K
HS19.11  817 KKLGKLWNIRKLSDVAK  833
```

Figure S9: Sequence comparison of the amidoligase domains Cj1438 and HS19.11. The overall sequence identity is ~61%.

```
HS19.11    1 MKTVGVVIPIYNVEKYLRECLDSVVNQTYKNLQVVLVNDGSTDENSLNIAKEYTLKDERF  60
             MKTVGVVIPIYNVEKYLRECLDSVVNQTYKNLQVVLVNDGSTDENSLNIAKEYTLKDERF
HS19.08    1 MKTVGVVIPIYNVEKYLRECLDSVVNQTYKNLQVVLVNDGSTDENSLNIAKEYTLKDERF  60

HS19.11   61 ILFDKENGGQSTARNVGIEFFSKEYDFKNITQELKENSLVEFKLDNEDNPYNIYKIYKSS  120
             ILFDKENGGQSTARNVGIEFFSKEYDFKNITQELKENSLVEFKLDNEDNPYNIYKIYKSS
HS19.08   61 ILFDKENGGQSTARNVGIEFFSKEYDFKNITQELKENSLVEFKLDNEDNPYNIYKIYKSS  120

HS19.11  121 NFFKNKDELLNFKAPDIDYIIFLDSDDYWELNCIEECVPRMDGVEVVWFD-NKAFDYEIK  179
             NFFKNKDELLNFKAPDIDYIIFLDSDDYWELNCIEECVPRMDGVEVVWFD NK   YE
HS19.08  121 NFFKNKDELLNFKAPDIDYIIFLDSDDYWELNCIEECVPRMDGVEVVWFDYNKI--YEKD  178

HS19.11  180 TIYPTSK-TFMECFNYNIKNKQINGNTWFDE-CRKNNITSIWIAVMEMIDFAYLKTLKLK  237
              +    + T+  C+N  IK   I  + W D+ C       +W     MI F YL   K+K
HS19.08  179 CLEKKDEWTWFNCYNMGIKKDIIISDEWLDKYCNIQTFAFVWSG---MIAFNYLSNQKIK  235

HS19.11  238 FLDGVLYEDNLFGTLLFLNVKKLYVLDKKLYNNRIRANSTMCHDNNLSFENLAP-FFRIL  296
             FLD + ++D  FG ++F    K+ +L+KK+ N RIR+N+T      +  + + P +   L
HS19.08  236 FLDYIFHQDVYFGFMVFFKSNKISLLNKKIINYRIRSNATTLRQGKIGEQIILPKYLDFL  295

HS19.11  297 SNDFLDPYDAREYIKLHSWTCMT  319
             S   +    DA++Y  L SW+ M
HS19.08  296 SKFYNKNEDAKKYYSLFSWSKMV  318
```

Figure S10: Sequence comparison between the N-terminal domains of HS19.11 (UniProt id: Q5M6M2) and HS19.08 (UniProt id: Q5M6M5). These domains are now predicted to catalyze the transfer of D-GlcNAc from UDP-D-GlcNAc to the serinol amide of D-glucuronic in the biosynthesis of the CPS of HS:19. The overall sequence identity is 67%.

REFERENCES:

1. Snitynsky, R.B., Lowary, T.L. Synthesis of Nitrogen-Containing Furanose Sugar Nucleotide for Use as Enzymatic Probes. *Org. Lett.* **2014**, *16*, 212-215.
2. Chen, W.-J., Han, Sh-B., Xie, Zh-B., Huang, H-Sh., Jiang, D-H., Gong, Sh-Sh., Sun. Q. Efficient Synthesis of UDP-Furanoses via 4,5-Dicyanoimidazole (DCI)-Promoted Coupling of Furanosyl-1-Phosphates with Uridine Phosphoropiperidate. *Molecules*, **2019**, *24*, 655.
3. Zhang, W., Hu, X., Carmichael, I., Seriann, A. S. Methyl [^{13}C]-Gluco-pyranosiduronic Acids: Effect of COOH Ionization and Exocyclic Structure on NMR Spin-Couplings. *J. Org. Chem.*, **2012**, *21*, 9521-9534.
4. Puchner, C., Eixelsberger, T. , Nidetzky B. , Brecker, L. Binding pattern of intermediate UDP-4-keto-xylose to Human UDP-xylose Synthase: Synthesis and STD NMR of Model Keto-saccharides. *Carbohydrate Research*, (**2017**, *437*, 50-58.
5. Wan, I., Witte, M. D., & Minnaard, A. J. From d- to l-Monosaccharide Derivatives via Photodecarboxylation-Alkylation. *Org. Lett.* **2019**, *21*, 7669– 7673.
6. Ágoston, K., Ágoston, Á., Dorgan, C. R., Fügedi, P. A New Method Testing the Orthogonality of Different Protecting Groups. *Carbohydrate Research*, **2015**, *418*, 98–103.
7. Riegert, A. S., Narindoshvili T., Platzer, N., Raushel, F. M. Functional Characterization of a HAD Phosphatase Involved in Capsular Polysaccharide Biosynthesis in *Campylobacter jejuni*. *Biochemistry*, **2022**, *62*, 3012-3019.
